# CLIP-170 is essential for MTOC repositioning during T cell activation by regulating dynein localisation on the cell surface

**DOI:** 10.1038/s41598-018-35593-z

**Published:** 2018-11-28

**Authors:** Wei Ming Lim, Yuma Ito, Kumiko Sakata-Sogawa, Makio Tokunaga

**Affiliations:** School of Life Science and Technology, Tokyo Institute of Technology, Nagatsuta-cho, Midori, Yokohama, Kanagawa 226-8501 Japan

## Abstract

The microtubule-organizing centre (MTOC) is repositioned to the centre of the contacted cell surface, the immunological synapse, during T cell activation. However, our understanding of its molecular mechanism remains limited. Here, we found that the microtubule plus-end tracking cytoplasmic linker protein 170 (CLIP-170) plays a novel role in MTOC repositioning using fluorescence imaging. Inhibition of CLIP-170 phosphorylation impaired both MTOC repositioning and interleukin-2 (IL-2) expression. T cell stimulation induced some fraction of dynein to colocalise with CLIP-170 and undergo plus-end tracking. Concurrently, it increased dynein in minus-end-directed movement. It also increased dynein relocation to the centre of the contact surface. Dynein not colocalised with CLIP-170 showed both an immobile state and minus-end-directed movement at a velocity in good agreement with the velocity of MTOC repositioning, which suggests that dynein at the immunological synapse may pull the microtubules and the MTOC. Although CLIP-170 is phosphorylated by AMP-activated protein kinase (AMPK) irrespective of stimulation, phosphorylated CLIP-170 is essential for dynein recruitment to plus-end tracking and for dynein relocation. This indicates that dynein relocation results from coexistence of plus-end- and minus-end-directed translocation. In conclusion, CLIP-170 plays an indispensable role in MTOC repositioning and full activation of T cells by regulating dynein localisation.

## Introduction

T cell activation is an essential step of the immune response. It is initiated by the recognition of the specific antigen displayed on the surface of an antigen-presenting cell (APC). The T cell receptor (TCR)/CD3 complex, composed of TCR subunits and CD3 subunits, recognizes antigenic peptides presented by major histocompatibility complex (MHC) molecules. This activation triggers the immune response in T cells, including cytokine production such as interleukin 2 (IL-2), and the dynamic reorganization of signalling molecules, as well as reorganization of actin and microtubule cytoskeletons. At the interface between the T cell and the APC, TCR signalling and related molecules are reorganized to the immunological synapse^1,2^, where the initial stages of the signaling cascade are spatiotemporally controlled on TCR/CD3 microclusters^3^. At almost the same time, MTOC undergoes dynamic repositioning and is moved to the immunological synapse^4–9^, where secretory vesicles are accumulated to allow focused secretion against the target cell^10,11^.

As for the driving motive force of MTOC movements, several lines of evidence have shown the involvement of cytoplasmic dynein, the major microtubule minus-end-directed motor protein, in MTOC repositioning^6–9,12,13^. Imaging of microtubules showed that the MTOC was pulled by microtubules, suggesting that dynein drives MTOC repositioning in T cells^6–9^. Depletion of dynein using small interfering RNA (siRNA) or inhibition of dynein activity with ciliobrevin was shown to prevent MTOC repositioning^7,9^.

Cytoplasmic dynein is involved in a variety of cellular functions, and its motor activity is regulated spatiotemporally by its interaction with a variety of regulatory proteins^14–16^. Dynein is a 1.4 MDa protein consisting of two copies of six different subunits, and this elaborate structure enables dynein to have a variety of activity. Recent studies with recombinant human dynein have unravelled the mechanism underlying its multimodal motor activities: auto-inhibited (dynein alone), weakly processive (dynein alone) and highly processive (dynein/dynactin/cargo-specific adaptor protein complex) using single-molecule techniques^17–21^, X-ray crystallography^22^ and cryo-electron microscopy^23^.

Given that dynein is anchored at the immunological synapse, its processive activity could pull on the microtubules. A candidate for the anchor is a dynein-binding protein, nuclear distribution E homolog 1 (NDE1), which functions to associate dynein with membranes^24^. NDE1 accumulates at the immunological synapse, whereas NDE-like 1 (NDEL1), a NDE1 homologue, does not^25^. Furthermore, knockdown of NDE1 in T cells were shown to inhibit MTOC translocation^25^.

Two mechanisms for targeting dynein to the plus end are known^26^. First, a subset of plus-end tracking proteins ( +TIPs), such as +TIP end-binding protein EB1, CLIP-170 and dynactin, recruits dynein to the plus-end^27,28^. Second, kinesin motor proteins complexed with CLIP-170 transport dynein-Lis1 complexes along microtubules to the plus end, and EB1 mediates loading of kinesin-CLIP-170 complexes onto microtubules^29,30^.

CLIP-170^31,32^, the key molecule in targeting dynein to the plus end, binds microtubules via EB1^33^. CLIP-170 contains two N-terminal CAP-Gly (cytoskeleton-associated protein glycine-rich) domains acting as the binding site for EB1, a central long coiled-coil dimerization domain, followed by tandem C-terminal Zn^2+^ knuckle domains, and an ETF motif ^34^. Dynactin and Lis1 competitively bind to the C-terminal domains of CLIP-170^35^. CLIP-170 is also responsible for the regulation of microtubule dynamics. CLIP-170 phosphorylated by AMP-activated protein kinase (AMPK) rapidly dissociates from the microtubule and promotes efficient microtubule polymerization^36^. As depletion of CLIP-170 was reported to block MTOC repositioning in B cells^12^, CLIP-170 is likely responsible for MTOC repositioning during B cell activation. However, the role of CLIP-170 on MTOC repositioning and how dynein is translocated to the immunological synapse in T cells have not been uncovered.

In this study, we focus on the role of CLIP-170 in MTOC repositioning and the interaction with dynein involved in this process during T cell activation. First, using fluorescence microscopy, we dissect MTOC repositioning into two directions, parallel and perpendicular to the contact surface. We show CLIP-170 and its phosphorylation is critical to the MTOC centreing. Then, using simultaneous dual-colour fluorescence imaging of CLIP-170, dynein and/or dynactin, we examine their colocalisation and motility states. We discover that dynein relocation to the immunological synapse relies on coexistence of plus-end- and minus-end-directed translocation due to CLIP-170 and T cell stimulation.

## Results

### CLIP-170 regulates MTOC repositioning and T cell activation

To visualise and quantify molecular interactions and dynamics of proteins underlying MTOC repositioning, we used a simultaneous dual-colour fluorescence microscope equipped with an illumination system enabling switching among total internal reflection fluorescence (TIRF), highly inclined and laminated optical sheet (HILO), and epi-fluorescence microscopy^37,38^. Jurkat T cells were costimulated with anti-CD3ε and anti-CD28 antibodies coated on glass bottom dishes. This costimulation induces full activation of T cell signalling^39^. In control experiments, T cells remained unstimulated on glass-bottom dishes coated with an anti-CD45 antibody. The full activation of Jurkat T cells by costimulation was confirmed by IL-2 expression quantification (Fig. S1).

**Figure 1:**
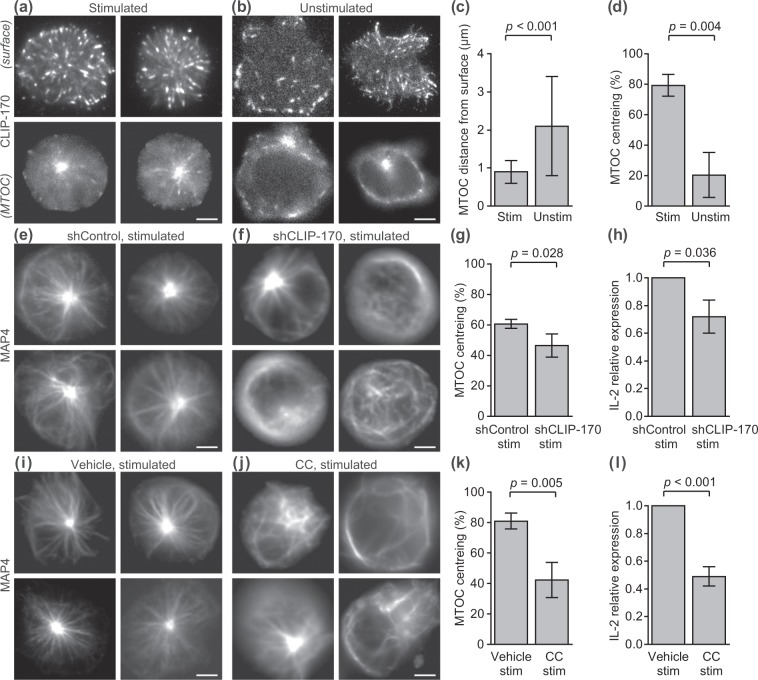
CLIP-170 regulates MTOC repositioning and full activation of T cells. (**a–d**) MTOC repositioning is composed of transpositions in two directions, perpendicular and parallel to the contact surface. Fluorescence live-cell imaging of CLIP-170-TagRFP-T (**a,b**) expressed in Jurkat T cells, visualised by TIRF at the surface of contact (top) and by epi-fluorescence microscopy at the plane containing MTOC (bottom), stimulated with the anti-CD3ε/anti-CD28 antibodies (**a**) and unstimulated with the control anti-CD45 antibody (**b**) coated glass bottom dishes. Perpendicular transposition quantified as the MTOC distance from the contact surface (**c**), and parallel transposition quantified as MTOC centreing, the fraction of cells whose MTOC was positioned at the centre region (**d**), analysed using the fluorescence images. (**e-h**) CLIP-170 knockdown impairs MTOC repositioning and full activation of T cells upon stimulation. MTOC localisation and microtubule frameworks visualised by epi-fluorescence live-cell microscopy using TagRFP-T-MAP4 (**e**,**f**) in stimulated Jurkat cells expressing control shRNAs (**e**) and CLIP-170 shRNAs (**f**). MTOC centreing (**g**), and the relative expression of IL-2 (**h**) quantified by qPCR in CLIP-170 shRNA-knockdown or control shRNA-expressing Jurkat cells stimulated on dishes. (**i–l**) The AMPK inhibitor compound C (CC) impairs MTOC repositioning and full activation of T cells upon stimulation. MTOC localisation and frameworks visualised in the same manner as in panels e and f (**i, j**) in the presence of vehicle alone (0.2% DMSO, **i**) or 20 μM compound C (**j**) in stimulated Jurkat cells. MTOC centreing (**k**), and the relative expression of IL-2 in the presence of vehicle or 20 μM compound C (**l**) in stimulated Jurkat cells. Cells were stimulated at 37 °C for 20 min with the coated antibodies, followed by imaging at 37 °C in all observations. Bars, 5 μm. Data are means ± standard deviation (SD). Source data, Tables S1 and S2. *p*, *p*-values; NS, not significant; stim, stimulated; unstim, unstimulated.

We first visualised microtubules using CLIP-170-TagRFP-T. As CLIP-170 binds only at the growing end of microtubule, we clearly visualized dynamic movement of microtubules as characteristic comet-like structures of CLIP-170. MTOCs were visualised by epi-fluorescence microscopy at a plane where the bright spot of MTOC was focused (Fig. 1a and b bottom). Imaging at the cell surface of contact by TIRF was used to measure the distance of the MTOC from the contact surface (Fig. 1a and b top).

Two quantities were measured to dissect MTOC repositioning into two directions, perpendicular and parallel to the contact surface (Fig. 1c and d, Table S1): (perpendicular direction) MTOC distance from the contact surface, calculated from the focusing shift and corrected for the refraction at the contact surface^38^; (parallel direction) MTOC centreing fraction, quantified as the fraction of cells whose MTOC was positioned at the centre region (i.e. the immunological synapse). The centre and periphery region was defined by dividing the cell surface by an ellipse with half the diameter of the cell of interest (Fig. S2). Comparison between the stimulated and unstimulated Jurkat cells showed that MTOC repositioning is composed of both transpositions: perpendicular transposition closer to the contact surface (Fig. 1c) and parallel transposition into the centre region (Fig. 1d).

To assess the functional contribution of CLIP-170 to MTOC repositioning, we performed CLIP-170 knockdown experiments. Vectors carrying shRNA targeting CLIP-170 with tandemly arranged EmGFP was constructed and transfected into Jurkat cells together with TagRFP-T-MAP4, which was used for visualisation of microtubules and MTOCs. Knockdown efficiency of shRNA used for live cell imaging was shown in Fig. S3. Only knockdown cells were observed using simultaneously expressed EmGFP. Fluorescence images of TagRFP-T-MAP4 in CLIP-170 knockdown cells stimulated with the coated anti-CD3ε/anti-CD28 antibodies showed disturbed microtubule frameworks and decentred the MTOC position (Fig. 1e and f). The MTOC centreing fraction in CLIP-170 knockdown cells was significantly decreased compared with wild-type cells (*p* = 0.028) (Fig. 1g, Table S2), which was consistent with the results of the previous reports on B cells^12^. Further, the degree of T cell activation was assessed by IL-2 expression, which was quantified by qPCR (quantitative real-time PCR). The relative IL-2 expression in CLIP-170 knockdown cells decreased significantly compared to that in the wild-type cells (*p* = 0.036) (Fig. 1h, Table S2). It is noteworthy that the presence of endogenous CLIP-170 counteracts the knockdown effect. Taken together, CLIP-170 knockdown impaired MTOC repositioning and full activation of T cells upon stimulation.

Next, we assessed the functional connectivity of CLIP-170 phosphorylation in MTOC repositioning. CLIP-170 is phosphorylated by AMPK^36^, and AMPK mediates IL-2 expression upon T cell stimulation^40^. Fluorescence imaging of MTOCs and microtubules using TagRFP-T-MAP4 showed that an AMPK inhibitor, compound C^41^, also disturbed microtubule frameworks and decentred the MTOC position (Fig. 1i and j). The MTOC centreing fraction of cells in the presence of compound C was significantly decreased compared with that in the absence of this compound (*p* = 0.005) (Fig. 1k, Table S2). The relative expression of IL-2 in the presence of compound C decreased significantly compared to that in the absence of this compound (*p* < 0.001) (Fig. 1l, Table S2). In contrast, compound C had no effect on TCR/CD3 microcluster formation upon stimulation (Fig. S4), indicating that CLIP-170 phosphorylation by AMPK is not involved in TCR signalling interactions during the initial stages of the activation. These results indicate that phosphorylation of CLIP-170 mediates both MTOC repositioning and full activation of T cells upon stimulation.

### CLIP-170 S312 phosphorylation is responsible for MTOC repositioning and T cell activation

To clarify the roles of CLIP-170 phosphorylation in MTOC repositioning, we generated two human CLIP-170 mutants with substitutions at Ser-312, which is the target residue of AMPK^36^, a phosphomimetic S312D mutant and a phosphodeficient S312A mutant.

**Figure 2:**
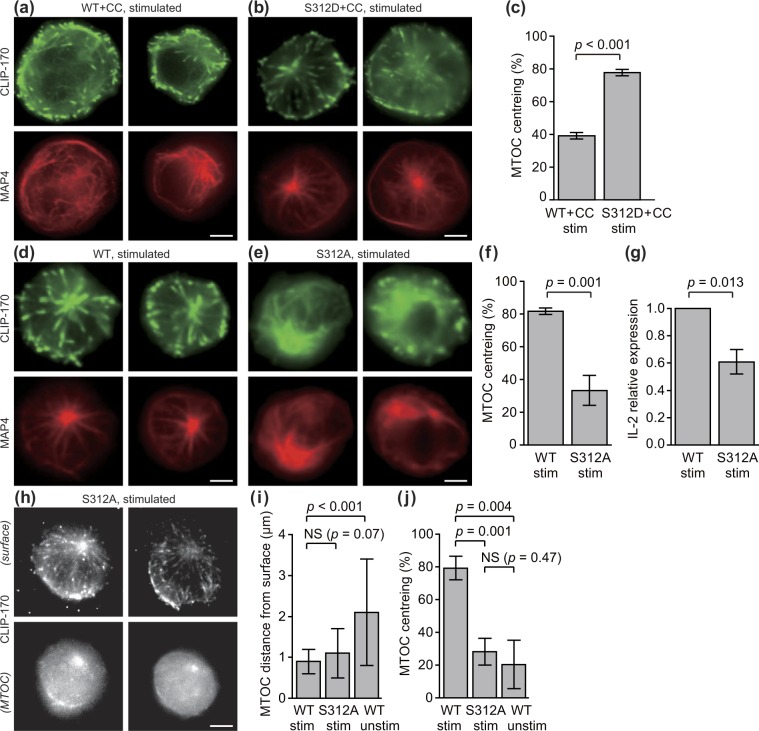
Both CLIP-170 phosphorylation and T cell stimulation are essential for MTOC repositioning and full activation of T cells. (**a**–**g**) A phosphodeficient S312A mutant of CLIP-170 impairs MTOC repositioning and full activation, and a phosphomimetic S312D mutant does not. Dual-colour epi-fluorescence live-cell imaging (**a**,**b**,**d** and **e**) at the contact surface in stimulated Jurkat cells using combinations of TagRFP-T-MAP4 (red, bottom) with C-terminal mEGFP-tagged proteins (green, top): wild-type CLIP-170 in the presence of 20 μM compound C (**a**), phosphomimetic S312D mutant in the presence of 20 μM compound C (**b**), wild-type CLIP-170 (**d**) and phosphodeficient S312A mutant (**e**). MTOC centreing (**c**,**f**) in Jurkat cells expressing wild-type- or S312D-mEGFP in the presence of 20 μM compound C (**c**) and wild-type or S312A-mEGFP (**f**). The relative expression of IL-2 (**g**) in Jurkat cells expressing wild-type or S312A CLIP-170. (**h–j**) MTOC centreing requires phosphorylated CLIP-170 at Ser-312, while MTOC distance from the surface is caused solely by stimulation. TIRF live-cell imaging of S312A-TagRFP-T (**h**) at the contact surface (top) and by epi-fluorescence at the plane containing MTOC (bottom) in stimulated Jurkat cells. MTOC distance from the surface (**i**), and MTOC centreing (**j**), quantified using the images. In panels i and j, the data in Fig. 1c and d are re-plotted for comparison. Bars, 5 μm. Data are means ± SD. Source data, Tables S1 and S3.

At first, to confirm that S312 is phosphorylated, we imaged C-terminal mEGFP-tagged proteins of the wild-type CLIP-170, and the S312D mutant, co-expressed with TagRFP-T-MAP4 in Jurkat cells in the presence of compound C (Fig. 2a and b). As shown in Fig. 2c, S312D mutation rescued the impaired MTOC centreing caused by AMPK inhibition (Fig. 1k). It is noteworthy that stimulated S312D mutant exhibits the similar MTOC centreing as wild-type (Fig. 2d), with (Fig. 2b) or without (Fig. S5) compound C. This observation suggests that most of endogenous CLIP-170 was phosphorylated by AMPK.

Further, we checked the effect of phosphodeficient mutation in S312. mEGFP-tagged proteins of the wild-type CLIP-170 and S312A mutants co-expressed with TagRFP-T-MAP4 were imaged (Fig. 2d and e). The phosphodeficient S312A mutant CLIP-170 showed disturbed microtubule frameworks and decentred the MTOC position. The centreing fraction was largely and significantly decreased in the phosphodeficient S312A mutant (*p* = 0.001) (Fig. 2f, Table S3). The relative IL-2 expression in cells with the S312A mutant decreased significantly compared to the wild-type cells (*p* = 0.013) (Fig. 2g, Table S3). Thus the effect of CLIP-170 Ser-312 phosphorylation was clearly shown.

To check the difference between the roles of CLIP-170 phosphorylation and T cell stimulation, we quantified the MTOC distance and centreing fraction. Fluorescence images of S312A mutant CLIP-170-TagRFP-T in stimulated Jurkat cells (Fig. 2h) was quantified (Fig. 2i and j), and compared with those of wild-type CLIP-170-TagRFP-T in stimulated cells and unstimulated cells (Fig. 1a–d). The MTOC distance from the contact surface of the S312A mutant in stimulated cells (1.1 ± 0.6 μm) was not significantly different from that of wild-type CLIP-170 in stimulated cells (0.9 ± 0.3 μm, *p* = 0.07) (Fig. 2i, Table S1). In contrast, those of wild type CLIP-170 in stimulated cells and in unstimulated cells (2.1 ± 1.3 μm) showed a significant difference (*p* < 0.001). On the contrary, the MTOC centreing fraction of the S312A mutant in stimulated cells largely and significantly decreased compared with that of the wild-type in stimulated cells (*p* = 0.001), and was not significantly different from that of the wild-type in unstimulated cells (*p* = 0.47) (Fig. 2j, Table S1). These findings indicate that: (1) MTOC parallel transposition, centreing, requires phosphorylated CLIP-170 at Ser-312; (2) MTOC perpendicular transposition closer to the surface is caused solely by stimulation without CLIP-170 phosphorylation; and (3) consequently, MTOC repositioning and full activation of T cells require both CLIP-170 phosphorylation and T cell stimulation.

### CLIP-170 phosphorylation up-regulates plus-end dynamics but T cell stimulation does not

**Figure 3:**
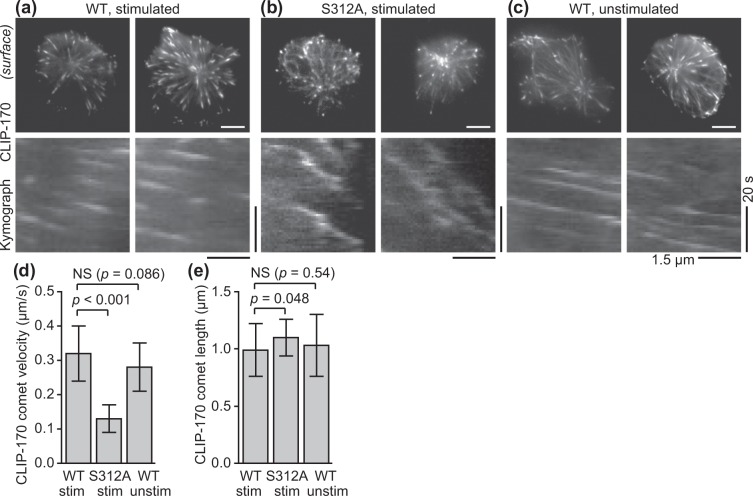
CLIP-170 phosphorylation up-regulates microtubule plus-end dynamics, but T cell stimulation does not. (**a**–**c**) TIRF live-cell images (top) and kymographs (bottom) of CLIP-170 comets and microtubule plus-end dynamics visualised using C-terminal mEGFP-tagged CLIP-170s: wild-type in stimulated cells (**a**), S312A mutant in stimulated cells (**b**), and wild-type in unstimulated cells (**c**) (see Movie S1). (**d**,**e**) The velocity (**d**) and the length (**e**) of CLIP-170 comets, quantified using the images. Bars in TIRF images, 5 μm; horizontal and vertical bars in kymographs, 1.5 μm and 20 s, respectively. Data are means ± SD. Source data, Table S4.

The effects of CLIP-170 phosphorylation at Ser-312 on microtubule plus-end dynamics were assessed. Microtubule plus-end dynamics and CLIP-170 comets were visualised using C-terminal mEGFP-tagged CLIP-170s. This was done for wild-type CLIP-170 in stimulated cells, phosphodeficient S312A mutant in stimulated cells, and wild-type in unstimulated cells (Fig. 3a–c upper, Movie S1). Microtubule plus-end dynamics was analysed using kymographs (Fig. 3a–c lower). The velocity of microtubule plus-end comet was calculated as the velocity of CLIP-170 comet transposition from the kymographs (Fig. 3d, Table S4). The CLIP-170 comet velocity of S312A mutant in stimulated cells decreased significantly compared with that of the wild-type in stimulated cells (*p* < 0.001). In contrast, that of the wild-type in unstimulated cells was not significantly different from that in stimulated cells (*p* = 0.09).

Meanwhile, the CLIP-170 comet length of S312A mutant in stimulated cells increased slightly compared with that of wild-types in stimulated cells (*p* = 0.048) (Fig. 3e, Table S4), meaning that unphosphorylated CLIP-170 stabilises the microtubule plus-end dynamics. The comet length of the wild-type in unstimulated cells again did not significantly differ from that in stimulated cells (*p* = 0.54).

Furthermore, in the phosphomimetic S312D mutant the effect of AMPK inhibition by compound C was disabled on both the CLIP-170 comet velocity and length (Fig. S6, Movie S2, Table S4). These results indicate that CLIP-170 phosphorylation up-regulates microtubule plus-end dynamics, consistent with previous reports^36^, but T cell stimulation does not affect this.

### Functional changes in dynamics and localisation of CLIP-170 and dynein

**Figure 4:**
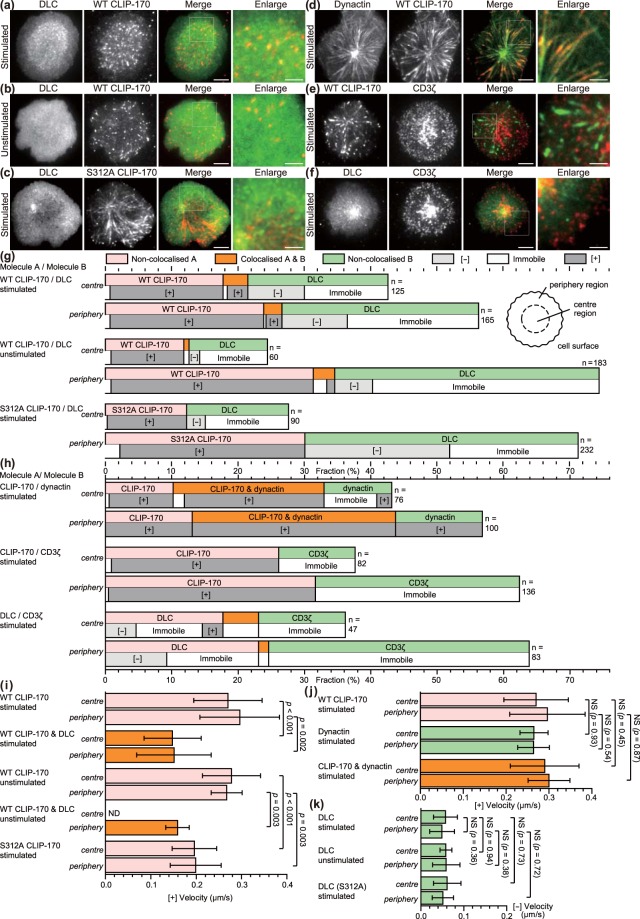
Both CLIP-170 phosphorylation and T cell stimulation are essential to relocate cytoplasmic dynein to the centre region. (**a–f**) Simultaneous dual-colour TIRF live-cell imaging showing the localisations of the clusters of CLIP-170, dynein, dynactin and the TCR/CD3 complex. Jurkat cells expressing dynein light chain (DLC)-mEGFP (left; green in merged images) and wild-type CLIP-170-TagRFP-T (2nd left; red in merged images) (**a,b**), DLC-mEGFP (left; green) and S312A mutant CLIP-170-TagRFP-T (2nd left; red) (**c**), dynactin-mEGFP (left; green) and CLIP-170-TagRFP-T (2nd left; red) (**d**), CLIP-170-mEGFP (left; green) and CD3ζ-TagRFP-T (2nd left; red) (**e**), DLC-mEGFP (left; green) and CD3ζ-TagRFP-T (2nd left; red) (**f**) were stimulated with the anti-CD3ε/anti-CD28 antibodies (**a**,**c**–**f**) or unstimulated with the control anti-CD45 antibody (**b**) coated on glass bottom dishes. The boxed regions in the merged images are enlarged (right). Bars for images (left, 2nd left, merged) and enlarged images are 5 μm and 2 μm, respectively. (**g**) Coexistence of plus-end- and minus-end-directed dynein at the centre, and increased dynein relocation to the centre requires both stimulation and CLIP-170 phosphorylation. Fractions of colocalisation between clusters of the two molecules (upper) at the centre or periphery region analysed using the images in panels a-c. Note that the total of all the fractions at the centre and periphery is 100%. The fractions are further classified by translocation (lower): plus-end- or minus-end-directed, or immobile. (**h**) Colocalisation and translocation of CLIP-170 and dynactin, CLIP-170 and the TCR/CD3 complex, and dynein and the TCR/CD3 complex are shown as the same as in panel g analysed using the images in panels d-f. (**i**,**j**) The velocities of plus-end tracking of the clusters quantified using the images in panels a-c and d-f corresponding to panels g and h, respectively. The velocities of wild-type CLIP-170 were re-plotted in panel j from panel i for comparison. See Table S5 for details. (**k**) The velocities of minus-end-directed movement of non-colocalised dynein clusters, quantified using the images in panel a-c corresponding to panel g. DLC (S312A), using the images of pair S312/DLC. Data are means ± SD. Source data for panels i-k, Table S5.

We investigated the functional relation between CLIP-170 phosphorylation and T cell stimulation. Using simultaneous dual-colour TIRF live-cell imaging, we visualised colocalisations and movement of proteins selected from among those potentially interacting with CLIP-170, specifically the dynein light chain (DLC, a subunit of dynein), dynactin, and CD3ζ (a subunit of the TCR/CD3 complex) (Fig. 4a–f, Movie S3–6). Jurkat cells coexpressing C-terminal mEGFP-tagged protein and C-terminal TagRFP-T-tagged counter-protein were stimulated with the anti-CD3ε/anti-CD28 antibodies or remained unstimulated with the control anti-CD45 antibody coated on glass bottom dishes.

When we compare the distribution of dynein in Fig. 4a–c, stimulated wild type-CLIP-170 cells (Fig. 4a) show dynein clusters accumulated in the inner area. CLIP-170 also localized in the same inner area, suggesting broad colocalisation. In case of stimulated S312A mutant (Fig. 4c), dynein is not accumulated in the inner area. The dynein cluster in the unstimulated cell (Fig. 4b) distributes more diffusely. It is due to the higher concentration of the non-clustered dynein. These observations suggest that dynein molecules are anchored at the cell surface of the central region after T cell stimulation.

Using the images in Fig. 4a–f, the fractions of colocalised and non-colocalised clusters and translocation velocities of clusters were quantified separately at the centre or periphery region (Fig. 4g–k). The cluster fraction was calculated as the percentage of all the cluster fractions located at either the centre or the periphery (total at both regions = 100%).

First, colocalisation and movement of CLIP-170 and dynein were examined (Fig. 4g). Comparison among the data from the wild-type CLIP-170 and dynein in stimulated cells, those in unstimulated cells, and the S312A mutant and dynein in stimulated cells showed that: (1) plus-end-directed dynein is only observed in colocalised dynein with CLIP-170; (2) that is produced by stimulation, especially at the centre; (3) minus-end-directed dynein is increased by stimulation, especially at the centre; (4) colocalised dynein with CLIP-170 requires CLIP-170 phosphorylation; (5) dynein relocation to the centre was largely increased by stimulation and by CLIP-170 phosphorylation.

Accordingly, coexistence of plus-end- and minus-end-directed dynein at the centre requires both T cell stimulation and CLIP-170 phosphorylation, and increased dynein relocation to the centre also requires both the stimulation and CLIP-170 phosphorylation. These indicate that: (1) both the stimulation and CLIP-170 phosphorylation are essential for coexistence of plus-end- and minus-end-directed dynein at the centre region; (2) both plus-end- and minus-end-directed dynein at the centre is necessary for dynein relocation to the centre; and together with the result of Figs 1 and 2, (3) dynein relocation to the centre is responsible for MTOC repositioning and full activation of T cells.

Next, colocalisation and movement of CLIP-170 and other proteins were examined (Fig. 4h). The majority of CLIP-170 and dynactin clusters showed colocalisation, whereas CLIP-170 and TCR/CD3 clusters revealed no colocalisation, and dynein and TCR/CD3 clusters only showed a partial one.

Finally, the translocation velocities of the clusters were quantified using the dual-colour images (Fig. 4i–k). The velocities of plus-end tracking of non-colocalised wild-type CLIP-170 in both stimulated and unstimulated cells, those of non-colocalised dynactin, and those of colocalised CLIP-170 and dynactin, did not significantly differ both at the centre and at the periphery (average 0.28 ± 0.07 μm/s) (Fig. 4i and j, Table S5), corresponding well to the microtubule growth rate reported previously (17.9 ± 7.7 μm/min, equivalent to 0.30 ± 0.13 μm/s in LLCPK1 cells)^42^.

Meanwhile, those of colocalised wild-type CLIP-170 and dynein, and those of S312A mutant CLIP-170 were slower than those of non-colocalised wild-type CLIP-170 (Fig. 4i, Table S5). These findings indicate that colocalisation of CLIP-170 with dynactin has no effect on the translocation velocity, while colocalisation of CLIP-170 with dynein acts as a source of resistance to the plus-end tracking. Accordingly, the lifetime of the plus-end-directed dynein cluster colocalised with CLIP-170 was approximately 9 s, almost half of that of the minus-end-directed dynein cluster (Table S6).

The velocities of minus-end-directed movement of the clusters were quantified (Fig. 4k, Table S5). Those of dynein in both stimulated and unstimulated cells, and in cells coexpressed with S312A mutant CLIP-170 did not significantly differ both at the centre and at the periphery. The average velocity was 0.054 ± 0.028 μm/s (Table S5). It should be noted that all the cluster of minus-end-directed dynein were non-colocalised with CLIP-170. This velocity is in good accordance with the velocity of dynein measured using single molecule imaging (0.079 ± 0.011 μm/s)^20^ at the “weakly processive” state, in which dynein does not make complexes with dynactin and cargos^23^. It is also noteworthy that the velocity is also in good accordance with the velocity of MTOC repositioning, 3.26 ± 0.77 μm/min, equivalent to 0.054 ± 0.013 μm/s, reported previously^9^.

## Discussion

**Figure 5:**
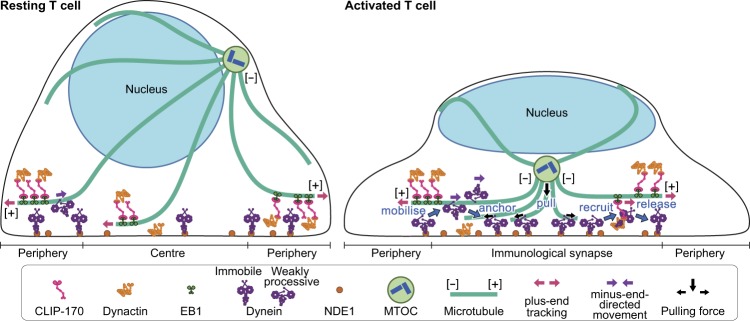
A schematic model for a key role of CLIP-170 in MTOC repositioning during T cell activation by regulating cytoplasmic dynein relocation to the immunological synapse. In resting Jurkat T cells, the majority of dynein is immobile on the contacted cell surface, and is located at the periphery region. T cell stimulation increases the fraction of dynein undergoing minus-end-directed motility (“mobilise”), which is a “weakly processive” state, i.e. not the highly processive complex with cargo and dynactin. Then, the dynein anchored to the surface after translocation less than one or two micrometres (“anchor”). Alongside this, stimulation induces some fraction of dynein to colocalise with CLIP-170 and dynactin, and follow the plus-end tracking (“recruit”). The recruited dynein has slower velocity and shorter lifetime, suggesting its interaction with membrane proteins, probably anchor proteins. After tracking of one or two micrometres, the dynein is released from the complex and anchored (“release”). As a result of coexistence of plus-end- and minus-end-directed translocation, dynein relocation increases to the centre region of the contact surface, the immunological synapse, where “anchored” dynein molecules are immobile and or weakly processive at a velocity in good agreement with the velocity of MTOC repositioning. “Anchored” and weakly processive dynein pulls the microtubules and the MTOC (“pull”), which causes MTOC repositioning near the immunological synapse and full activation of T cells. Phosphorylation of CLIP-170 is essential for dynein recruitment to the plus-end tracking and for dynein relocation.

In contrast to the importance of MTOC repositioning during T cell activation, the molecular mechanisms underlying this process remain unknown. In this study, we report a novel role of CLIP-170 in regulating dynein localization analysed by simultaneous dual-colour fluorescence live-cell microscopy. Phosphorylated CLIP-170 is essential for dynein recruitment to the plus-end tracking. T cell stimulation increases dynein in minus-end-directed movement. Both plus-end- and minus-end-directed translocation of dynein are necessary for dynein relocation to the immunological synapse. Based on these results, we propose a model of MTOC repositioning (Fig. 5).

There are several discussions on the motive force of MTOC repositioning: (1) the dynein-driven pulling mechanism, where dynein is anchored at the immunological synapse and its processive activity pulls on the microtubules; (2) a capture-shrinkage mechanism, where cortically bound dynein interacts with the plus end of a microtubule in a way as to couple the subsequent microtubule depolymerisation^13^; (3) a dynein-independent mechanism, an actin-dependent model, where microtubules are anchored to the peripheral F-actin ring and forces are exerted on microtubules by expansion of the F-actin ring, together with forces generated by cortex-associated dynein^12^. Our present findings support the dynein-driven pulling mechanism.

The rate of MTOC repositioning was reported to be 0.054 ± 0.013 μm/s (3.26 ± 0.77 μm/min)^9^. The present result of the average velocity 0.054 ± 0.028 μm/s of minus-end-directed dynein is in good accordance with the MTOC repositioning rate. Recently, using cryo-electron microscopy a structural study on activation mechanism of cytoplasmic dynein revealed that dynein, which does not form a complex with dynactin and cargo activator protein, remains in a “weakly processive” state^23^. A study using single-molecule assays reported that the *in vitro* velocity of dynein alone is 0.079 ± 0.011 μm/s^20^, which is also in good accordance with the present result. These multiple lines of findings support the dynein-driven pulling mechanism, and indicate that MTOCs undergoing repositioning during T cell activation is pulled by immobilised cytoplasmic dynein in the “weakly processive” state at the immunological synapse (Fig. 5, “pull”).

The role of CLIP-170 on MTOC repositioning during T cell activation has not been elucidated. Dissection of MTOC repositioning into the two directions has shed a new light on it. The imaging studies using CLIP-170 phosphosite mutants reveal that MTOC parallel transposition, centreing, requires both CLIP-170 phosphorylation and T cell stimulation, whereas MTOC perpendicular transposition is caused solely by stimulation without requiring CLIP-170 phosphorylation. In addition to this asymmetric joint role of CLIP-170 phosphorylation and T cell stimulation, we find one more difference in their role: CLIP-170 phosphorylation up-regulates microtubule plus-end dynamics, whereas T cell stimulation does not affect plus-end dynamics. These seemingly contradictory findings have been solved by the simultaneous dual-colour fluorescence live-cell microscopy as follows: colocalisation of dynein with CLIP-170 is caused solely by CLIP-170 phosphorylation, and is increased by T cell stimulation. Plus-end-directed dynein is only observed in colocalised dynein with CLIP-170. Thus, CLIP-170 phosphorylation regulates MTOC repositioning via dynein recruitment not via microtubule plus-end dynamics.

The simultaneous dual-colour fluorescence imaging and mobility analysis enabled us to study characteristics of interactions and dynamics of CLIP-170, dynein, dynactin and the TCR/CD3 complex. CLIP-170 alone or together with dynactin translocates to the plus-end at 0.28 ± 0.07 μm/s. This corresponds well with the rate of microtubule growth (0.30 ± 0.13 μm/s in LLCPK1 cells)^42^. Meanwhile, the plus-end-directed velocity of colocalised CLIP-170 and dynein is slower than that of non-colocalised CLIP-170, indicating that colocalisation of dynein with CLIP-170 acts as a source of resistance to the plus-end tracking. Additionally, the lifetime of the plus-end-directed dynein cluster colocalised with CLIP-170 is almost half of that of the minus-end-directed dynein cluster. These observations may also suggest that the colocalised dynein interacts with other proteins, such as anchor proteins immobilised to the cell membrane (Fig. 5, “recruit” and “release”).

Recently, the balance between the microtubule plus-end tracking and minus-end-directed motility of cytoplasmic dynein is receiving attention^16^. Here we have found that their coexistence is indispensable in the MTOC repositioning. Among the present findings, both CLIP-170 phosphorylation and T cell stimulation are required for: (1) plus-end-directed dynein, which is only observed in colocalised dynein with CLIP-170 (Fig. 5, “recruit”); (2) coexistence of plus-end- and minus-end-directed dynein at the centre region; (3) dynein relocation to the centre (Fig. 5, “mobilise” and “anchor”); (4) MTOC repositioning and full activation of T cells. These findings indicate that coexistence of plus-end- and minus-end-directed dynein is a key determinant of dynein relocation and MTOC repositioning.

This key determinant is generated by recruiting dynein to the complex with phosphorylated CLIP-170, and is increased by T cell stimulation (especially at the centre largely increased). This finding raises a question how dynein is recruited to plus-end tracking by phosphorylated CLIP-170. The recruitment could be due to phosphorylation of dynein caused by weakening of the anchoring to the cell surface of contact, since phosphorylation of dynein intermediate chain has been reported to reduce its interaction with NDEL1^43^, the homologue of NDE1, the anchor candidate^24,25^, with a high sequence similarity. Meanwhile, NDE1 and dynactin forms mutually exclusive complexes with the dynein intermediate chain^25^. Furthermore, two isoforms DCTN1A and DCTN1B of dynactin 1, the largest subunit of dynactin, have antagonistic functions on dynein activity: the tripartite complex of dynein-dynactin-cargo adaptor BICD2 containing DCTN1A exhibits highly processive movement; the complex of dynein-dynactin containing DCTN1B shows no apparent processive movement^21^. These findings suggest that the dynactin containing DCTN1B could function as a recruiter of dynein. The molecular mechanisms underlying the recruitment of dynein to plus-end tracking and the anchoring of dynein remain to be solved.

This work demonstrates a novel and indispensable role of CLIP-170 in MTOC repositioning and full activation of T cells. Coexistence of plus-end- and minus-end-directed dynein is generated via the joint role of CLIP-170 phosphorylation and T cell stimulation. This is essential for dynein relocation to the immunological synapse, where the immobilised dynein may pull the MTOC. We anticipate that the present findings shed new light on biological processes involving microtubule binding proteins and microtubule dynamics.

## Materials and Methods

### Plasmid constructions

Human full-length CLIP-170, DLC (dynein light chain LC8-type 1, DYNLL1) and dynactin (DCTN1) genes were cloned from Jurkat T cells (E6-1, ATCC TIB-152). Briefly, total RNA was isolated from Jurkat T cells using TRIzol Plus RNA Purification Kit (Invitrogen), and cDNA fragments were amplified by RT-PCR using the SuperScript III One-Step RT-PCR system (Takara, Japan) with Platinum Taq High Fidelity (Invitrogen). The cDNAs were subcloned into the modified mammalian expression vector pEGFP-N1 (Clontech) in which the EGFP was converted to monomeric EGFP (mEGFP, EGFP A206K)^44^ and a flexible linker encoding (GGGGS)_3_ was inserted between cDNA and mEGFP^45,46^. The CLIP-170 mutants (S312A and S312D) were generated by site-directed mutagenesis from the wild type CLIP-170 gene sequence. CD3ζ from CD3ζ-EGFP cDNA (a gift from Drs. T. Yokosuka and T. Saito, RIKEN, Yokohama, Japan)^47^, MAP4 from pCT-MAP4-GFP (System Bioscience) and CLIP-170 (wild-type, S312A & S312D) were fused to the N-terminus of TagRFP-T (modified from pTagRFP-C, Evrogen)^48^. Primers for cloning and mutagenesis are listed in Table S7.

### Cell culture and transfection

Jurkat T cells were maintained at 37 °C and 5% CO_2_ humidified atmosphere in RPMI-1640 (Gibco, USA) supplemented with 10% fetal bovine serum (Gibco), 2 mM glutamine, 50 U/ml penicillin and 50 μg/ml streptomycin (Gibco). DNA transfection was performed using the NEON electroporation system (Invitrogen) according to the manufacturer’s protocol. The electroporation condition was optimized at 5.0 × 10^5^ cells, 5 μg of DNA, 1200 V (pulse voltage) and 40 ms (pulse width).

### T-cell stimulation

For live cell imaging, 35-mm glass bottom dishes (MatTek) were incubated overnight at 4 °C with stimulatory or nonstimulatory antibody solution to adsorb antibodies on the surface. The stimulatory antibodies were 1 μg/ml mouse anti-human CD3ε antibody (HIT3a, BD Pharmingen) and 1 μg/ml mouse anti-human CD28 antibody (CD28.2, BD Pharmingen) solution, while the nonstimulatory one was 1 μg/ml mouse anti-human CD45 antibody (MEM-28, abcam) solution. Just before imaging, the antibody-coated glass bottom dishes were washed twice with PBS, then Jurkat T cells (1–2 × 10^4^ cells) were stimulated by incubation on the antibody-coated dishes at 37 °C for 20 min in an imaging medium (25 mM HEPES and MEM without phenol red, riboflavin, and folic acid). For qPCR experiments, cells were stimulated on the anti-CD3ε/anti-CD28 antibody-coated culture dishes at 37 °C for 24 hours.

### Quantitative real-time PCR

Total mRNA from stimulated Jurkat T cells was extracted using PureLink RNA Mini Kit (Life technologies). Relative expression of IL-2 was quantified by qPCR^49^. Quantification of IL-2 mRNA was performed on a Thermal Cycler Dice Real Time System II (Takara) using the One Step SYBR PrimeScript PLUS RT-PCR kit (Takara). The primer pairs are shown in Table S8. The PCR mixture contained 2 μl of extracted mRNA, 10 μl of One Step SYBR RT-PCR buffer 4 (2x), 1.2 μl of Takara Ex Taq HS Mix, 0.4 μl of PrimeScript PLUS RTase Mix, primer pairs (optimized to final concentration of 10 μM) and sterile water to a final reaction volume of 20 μl. The optimal cycling conditions were 5 min at 42 °C, 10 s at 95 °C; 40 cycles of 5 s at 95 °C, 30 s at 60 °C. All qPCR reactions were performed in triplicate. Data were normalized against β-Actin expression, and the relative expression of IL-2 was calculated using the comparative C_T_ method.

### Knockdown experiments

Knockdown experiments were performed using expression vectors for short hairpin RNAs (shRNA) targeting human CLIP-170 (Block-iT Pol II miR RNAi Expression Vector Kit, Thermo Fisher Scientific) and the NEON electroporation system (Thermo Fisher Scientific). The shRNA sequences were designed using BLOCK-iT RNAi Designer (Invitrogen, Table S9). The cloned CLIP-170 sequences from Jurkat T cell (shCLIP-170 #1) and the reference sequence database (NM_001247997.1, shCLIP-170 #2) were used as references. Prior to use in Jurkat T cells, the knockdown efficiency was confirmed using HeLa cells. The cells were cultured in DMEM supplemented with 10% fetal bovine serum. Transfection of HeLa cells with the knockdown vector was carried out using the Neon electroporation system. After incubation at 37 °C for 24 h, cells were selected with 20 μg/ml blasticidin for 24 h, then the RNA and protein were extracted for qPCR and immunoblotting analysis. The knockdown efficiency of shRNA against CLIP-170 was assessed by qPCR and immunoblotting (Supplemental Methods). According to the knockdown efficiency (Fig. S3), the sequence shCLIP170 #1 was used for knockdown experiments in Jurkat T-cells.

Jurkat cells were transfected with the vector carrying shRNA targeting shCLIP-170 #1 with tandemly arranged EmGFP. Simultaneously expressed EmGFP was used for selection of knockdown cells during live cell imaging.

### Live cell imaging

Cells were imaged using an inverted microscope (IX83, Olympus, Japan) equipped with custom-built TIRF and HILO microscope setup^37,38^. The microscope is equipped with an infinity-corrected objective (PlanApo 100x NA 1.45 oil TIRFM, Olympus) and two solid-state lasers (Sapphire 488-20 & Compass 561-50, Coherent, Japan) for the fluorescent illumination. The microscope optical filters were custom-ordered (Olympus) to include a dichroic mirror (DM488) and emission filters (Em 495-545 for EGFP, Em569-624 for TagRFP-T). The Jurkat T cells were imaged at 37 °C using temperature control system with a stage top incubator and an objective heater (IBC-IU2-YOP/-CB/-LH, MI-IBC-IU2, Tokai Hit, Japan). The dual-colour images were simultaneously captured at an approximately 80 nm/pixel magnification with two electron-multiplying charge-coupled device (EMCCD) cameras (C9100-13, Hamamatsu Photonics, Japan) controlled by AQUACOSMOS software (Hamamatsu Photonics) at a frame rate of 1 frame/s. The magnification difference, shift and rotation between the two colour images were corrected using ImageConverter (Olympus, Japan) based on Bicubic interpolation with two colour images of a 10-μm square lattice (Olympus) captured at the same time.

### MTOC repositioning analysis

Jurkat cells were imaged at 37 °C using an inverted microscope (IX71, Olympus) equipped with an infinity-corrected objective (PlanApo 100x NA 1.40 oil TIRFM, Olympus, Japan), optical filters (U-MCFPHQ, Olympus), and the temperature control system (Tokai Hit). GFP and RFP were excited with a 490-nm light-emitting diode (LED) and a 535-nm LED, respectively (pE-2, CoolLED). Fluorescence images were captured with a cooled CCD camera (C10600-10B, Hamamatsu Photonics) controlled by MetaMorph (Molecular Devices, Japan).

The distance between the MTOC and the cell surface was measured using the specimen focusing z-stage of the microscope. The corrected distance *z* was calculated from z-stage mechanical shift *z*_0_ as:

1 $$z={z}_{0}\frac{{n}_{{\rm{specimen}}}}{{n}_{{\rm{glass}}}},$$

where *n*_specimen_ is the refractive index of the specimen, and *n*_glass_ is that of the coverslip and immersion oil^38^. We used *n*_specimen_ of 1.37, that of the cytoplasm of Jurkat cells^50^, and *n*_glass_ of 1.52 that of BK7 and immersion oil.

MTOC centreing at the cell surface during T cell activation was quantified as a fraction of cells whose MTOC was positioned at the centre region. The “centre” and “periphery” regions were divided by an ellipse with a half diameter of the cell of interest (see Fig. S2 in detail).

### CLIP-170 comets velocity and length analysis

Kymographs were created using the Multi Kymograph plugin for ImageJ^51^. The path along a microtubule was traced manually with a segmental line, which was then used in turn to generate a linear intensity profile along the path, i.e., the axis of the microtubule for each frames. This linear intensity profile was represented as a row with a single pixel of the line width, and stacked vertically in temporal sequence from top to bottom. It yielded a kymograph as a two-dimensional image with pitches of 80 nm/pixel and 1 s/pixel along the horizontal space and vertical time axes.

The CLIP-170 comet velocity *v* was calculated as the slope of the straight lines in trajectories. The CLIP-170 comet length was calculated as follows: the beginning of the comet was determined as the point where fluorescence intensity (a.u.) encountered rapid rise; its end as the point where fluorescence intensity reached baseline^34^. A minimum of 30 comets were analysed for each data set.

### Colocalization and movement analysis

The colocalisation and movement directions of protein clusters were analysed using simultaneous dual-colour TIRF live-cell images by ImageConverter (Olympus Software Technology). Protein clusters were selected manually according to the criteria: the diameter is larger than 240 nm (3 pixels); the difference of the image intensity between the cluster and the surrounding is larger than the standard deviation of that of the surrounding. The protein clusters were categorized into “centre” or “periphery” by their localisation at the centre or periphery region, and into “microtubule plus-end-directed”, “microtubule minus-end-directed” or “immobile” by their movement directions. If the protein clusters of the two kinds of proteins in the dual-colour images were kept colocalised and involved in the same movement category during not less than three consecutive frames, they were counted as “colocalised”. A minimum of 100 cluster sets obtained from five cells were analysed for each data set.

## Electronic supplementary material

Video 1

Video 2

Video 3

Video 4

Video 5

Video 6

Supplementary Information
