# Supplementary material for: CLIP-170 is essential for MTOC repositioning during T cell activation by regulating dynein localisation on the cell surface
Source: Sci Rep. 2018 Nov 28;8:17447. doi: 10.1038/s41598-018-35593-z (PMC6261991; doi:10.1038/s41598-018-35593-z)
Supplement: Supplementary file 7 — Supplementary Information [file 41598_2018_35593_MOESM7_ESM.pdf]

Supplementary Information

CLIP-170 is essential for MTOC repositioning during T cell activation by regulating dynein localisation on the cell surface

Wei Ming Lim[1,2], Yuma Ito[1,2], Kumiko Sakata-Sogawa[1]* & Makio Tokunaga[1]*

[1] School of Life Science and Technology, Tokyo Institute of Technology, Nagatsuta-cho, Midori, Yokohama, Kanagawa 226-8501, Japan.

[2] These authors contributed equally to this work.

*Correspondence and requests for materials should be addressed to M.T. (email: mtoku@bio.titech.ac.jp) or K.S.-S. (email: ksogawa@hilo.bio.titech.ac.jp)

Supplementary information comprises:

Supplementary Methods
Supplementary Movie Legends
Supplementary Figures S1–S6
Supplementary Tables S1–S9

Supplementary Methods

Immunoblotting

Cells were rinsed twice with PBS and lysed in lysis buffer (2 mM EDTA, 10 mM Tris-HCl pH 7.5, 150 mM NaCl, 1% Nonidet P-40) containing protease inhibitors cocktail (0.1% Aprotinin, 3 µg/ml Antipain, 3 µg/ml Cymostatin, 10 µg/ml Leupeptin, 5 µg/ml Pepstatin A and 1 mM PMSF). After 20 min on ice, lysates were cleared by centrifugation at 20,000 × g for 10 min at 4°C. Protein concentration was measured using bicinchoninic acid protein assay reagent (Pierce). Equal amounts of protein were heated at 95°C for 10 min with 2 × SDS gel loading buffer, and resolved on 7.5% SDS-polyacrylamide gels (Bio-Rad) by electrophoresis at 50 V for 60 min followed by 75 V for 90 min on ice. The proteins were transferred to a polyvinylidene fluoride membrane (Merck Millipore) using TRANS-BLOT semidry transfer cell (Bio-Rad) at 8 mA for 40 min. Immunoblotting was performed using the iBind Western system (Thermo Fisher Scientific) according to manufacturer's instructions. The following antibodies were use: anti-CLIP-170 (ab134907, abcam), anti-GAPDH (sc-47724, Santa Cruz), HRP-conjugated anti-mouse IgG (G21040, Invitrogen) and HRP-conjugated anti-rabbit IgG (G21234, Invitrogen). The signal was detected using SuperSignal West Dura Extended Duration substrate (Pierce) and ImageQuant LAS-4000 mini imager (GE Healthcare).

Supplementary Movie Legends

Movie S1

TIRF live-cell imaging of CLIP-170 comets and microtubule plus-end dynamics, showing that CLIP-170 phosphorylation up-regulates plus-end dynamics but T cell stimulation does not. Jurkat cells expressing C-terminal mEGFP-tagged CLIP-170s were imaged: wild-type in stimulated cells (**left**), phosphodeficient S312A mutant in stimulated cells (**centre**), and wild-type in unstimulated cells (**right**) (see **Fig. 3**). Cells were stimulated with the anti-CD3ε/anti-CD28 antibodies or unstimulated with the control anti-CD45 antibody coated on glass bottom dishes at 37°C for 20 min, followed by imaging at 37°C in all observations. Images were captured at a frame rate of 1 frame/s, and displayed at 20 frames/s. Bar, 5 µm.

Movie S2

TIRF live-cell imaging of CLIP-170 comets and microtubule plus-end dynamics, showing that CLIP-170 phosphorylation at Ser-312 by AMPK up-regulates the microtubule plus-end dynamics. Jurkat cells expressing mEGFP-tagged CLIP-170s: wild-type in the presence of vehicle alone (0.2% DMSO, **left**) or 20 µM compound C

(AMPK inhibitor, **centre**), and phosphomimetic S312D mutant in the presence of 20 µM compound C (**right**) were stimulated with the anti-CD3ε/anti-CD28 antibodies coated on glass bottom dishes (see **Fig. S6**). Images were captured at a frame rate of 1 frame/s, and displayed at 20 frames/s. Bar, 5 µm.

Movie S3

Simultaneous dual-color TIRF live-cell imaging showing the localisations of CLIP-170 and dynein (see **Fig. 4a-c**). Jurkat cells expressing dynein light chain (DLC)-mEGFP (left; green in merged images) and wild-type CLIP-170-TagRFP-T (2nd left; red in merged images) (**top, middle**), DLC-mEGFP (left; green) and S312A mutant CLIP-170-TagRFP-T (2nd left; red) (**bottom**) were stimulated with the anti-CD3ε/anti-CD28 antibodies (**top, bottom**) or unstimulated with the control anti-CD45 antibody (**middle**) coated on glass bottom dishes. The boxed regions in the merged images are enlarged (right). Bars for images (left, 2nd left, merged) and enlarged images are 5 µm and 2 µm, respectively. Images were captured at a frame rate of 1 frame/s, and displayed at 20 frames/s.

Movie S4

Simultaneous dual-color TIRF live-cell imaging showing the localisations of dynactin and CLIP-170 (see **Fig. 4d**). Jurkat cells expressing dynactin-mEGFP (left; green) and CLIP-170-TagRFP-T (2nd left; red) were stimulated with the anti-CD3ε/anti-CD28 antibodies coated on glass bottom dishes. The boxed region in the merged image is enlarged (right). Bars for images (left, 2nd left, merged) and enlarged images are 5 µm and 2 µm, respectively. Images were captured at a frame rate of 1 frame/s, and displayed at 20 frames/s.

Movie S5

Simultaneous dual-color TIRF live-cell imaging showing the localisations of CLIP-170 and the TCR/CD3 complex (see **Fig. 4e**). Jurkat cells expressing CLIP-170-mEGFP (left; green) and CD3ζ-TagRFP-T (2nd left; red) were stimulated with the anti-CD3ε/anti-CD28 antibodies coated on glass bottom dishes. The boxed region in the merged image is enlarged (right). Bars for images (left, 2nd left, merged) and enlarged images are 5 µm and 2 µm, respectively. Images were captured at a frame rate of 1 frame/s, and displayed at 20 frames/s.

Movie S6

Simultaneous dual-color TIRF live-cell imaging showing the localisations of dynein and the TCR/CD3 complex (see **Fig. 4f**). Jurkat cells expressing DLC-mEGFP (left; green) and CD3ζ-TagRFP-T (2nd left; red) were stimulated with the

anti-CD3ε/anti-CD28 antibodies coated on glass bottom dishes. The boxed region in the merged image is enlarged (right). Bars for images (left, 2nd left, merged) and enlarged images are 5 μm and 2 μm, respectively. Images were captured at a frame rate of 1 frame/s, and displayed at 20 frames/s.

Supplementary Figures

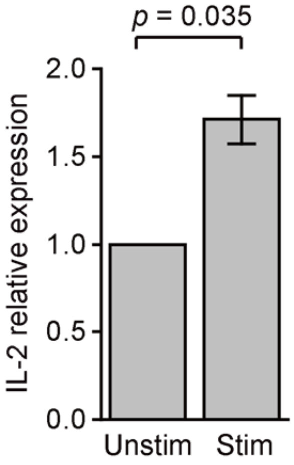

Figure S1. Real-time RT-PCR analysis of IL-2 gene expression in stimulated Jurkat cells. Cells were incubated on the anti-CD3ε/anti-CD28 (stimulatory) or anti-CD45 (nonstimulatory) antibody coated dishes for 24 h, then the total RNA was extracted and subjected to qPCR analysis of IL-2. Data shown are the mean fold change ± SD of three independent experiments.

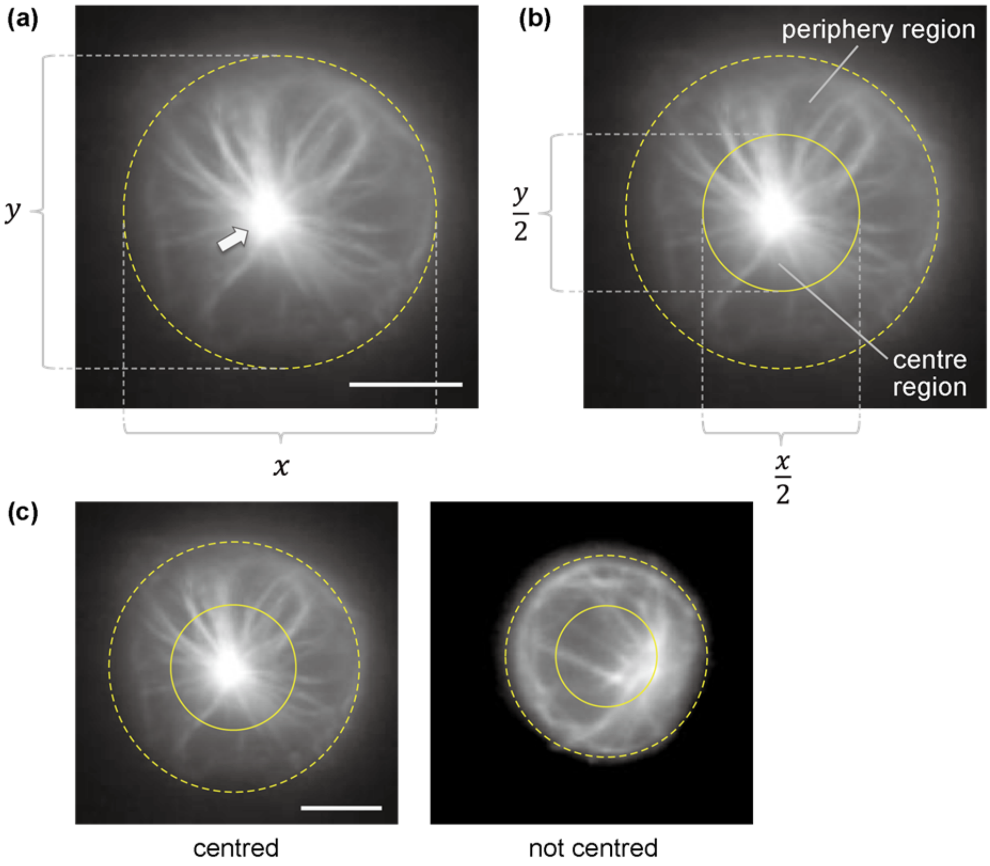

Figure S2. Quantification of MTOC centreing. Centreing was quantified as a fraction of cells whose MTOC positioned to the centre region. The "centre" and "periphery" regions were divided by an ellipse with a half diameter of the cell of interest. (**a**) The diameters of the cell were determined by approximating the cell contour to an ellipse with diameters x and y in the x- and y-direction, respectively. The arrow indicates MTOC. (**b**) The boundary between the "centre" and "periphery" region was defined as an ellipse with the half diameters $x/2$ and $y/2$ in the x- and y-direction, respectively. (**c**) MTOCs were scored as "centred" if they reside at the "centre" region. They were scored as "not centred" if they reside at the "periphery" region, and were scored also as "not centred" if they resides on the boundary or they are not clearly imaged. Scale bar, 5 μm.

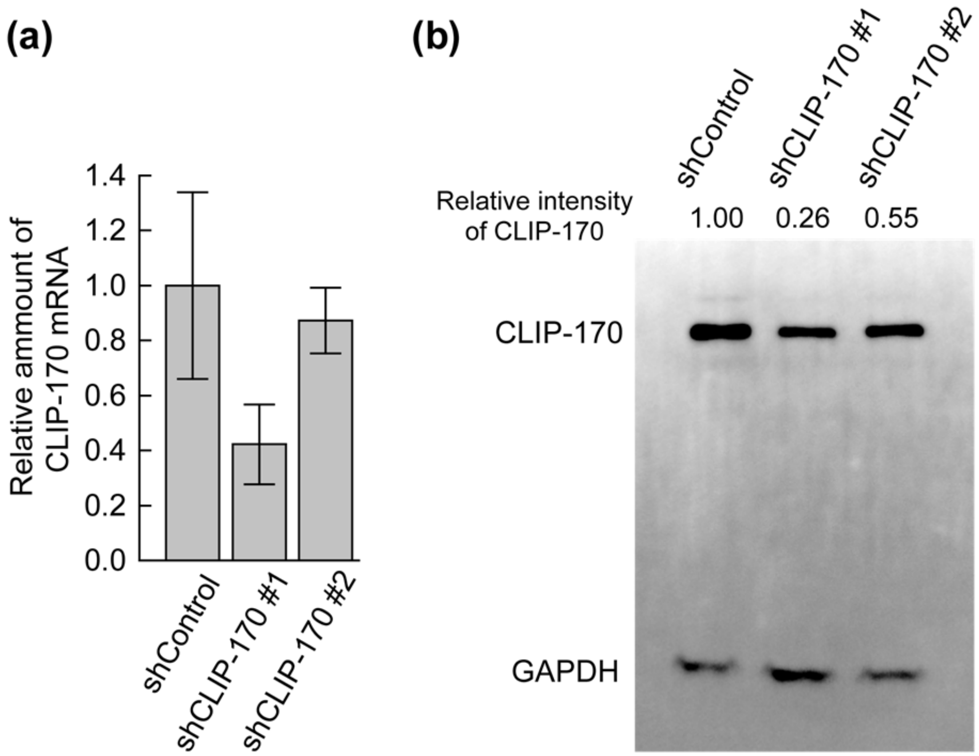

Figure S3. The knockdown efficiency of shCLIP-170. The knockdown plasmids were transfected into HeLa cells and selected with blasticidin (20 mg/mL, 24-48 h after transfection), then the total RNA and protein were extracted. The cloned CLIP-170 sequences from Jurkat T cell (shCLIP-170 #1) and the reference sequence database (NM_001247997.1, shCLIP-170 #2) were used as references in shRNA design. (**a**) Reduced amount of CLIP-170 mRNA was confirmed with qPCR. The data indicate the relative mRNA amount ± SD of technical triplicates. (**b**) Protein expression of CLIP-170 was assessed by immunoblotting analysis. Values above the blot represent the relative band intensity of CLIP-170 compared to shControl after normalization to that of GAPDH. The sequence of shCLIP170 #1 was used for knockdown experiments in Jurkat T-cells, since it showed higher knockdown efficiency.

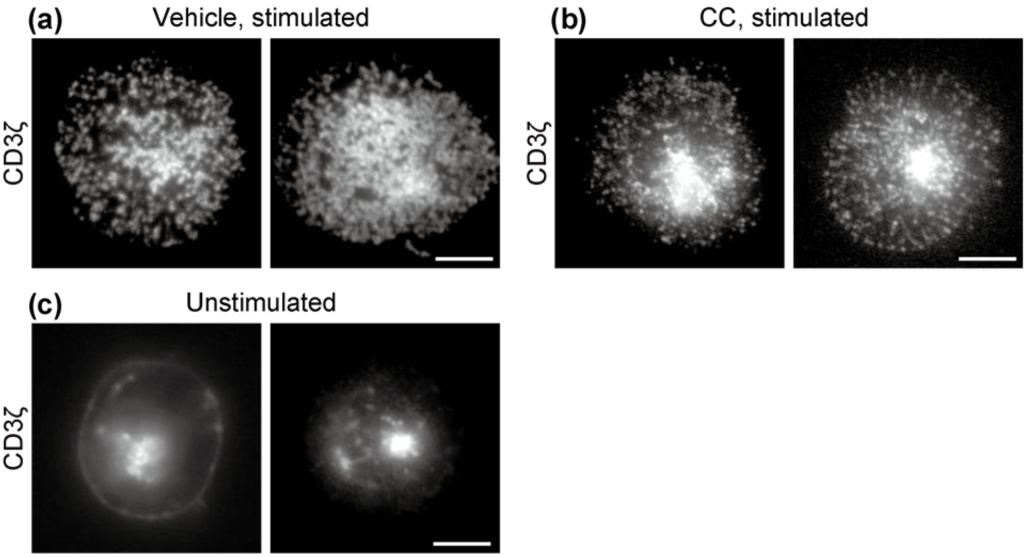

Figure S4. Compound C does not inhibit TCR/CD3 microcluster formation. TIRF live-cell imaging of the TCR/CD3 complex at the contact surface in Jurkat T cells expressing CD3ζ-TagRFP-T in the presence of vehicle alone (0.2% DMSO, **a**) or 20 μM compound C (**b**) stimulated with the anti-CD3ε/anti-CD28 antibodies coated on glass bottom dishes, and in unstimulated Jurkat cells expressing CD3ζ-TagRFP-T (**c**) incubated on the control anti-CD45 antibody-coated dishes. Cells were stimulated at 37°C for 20 min with the coated antibodies, followed by imaging at 37°C in all experiments. Scale bar, 5 μm.

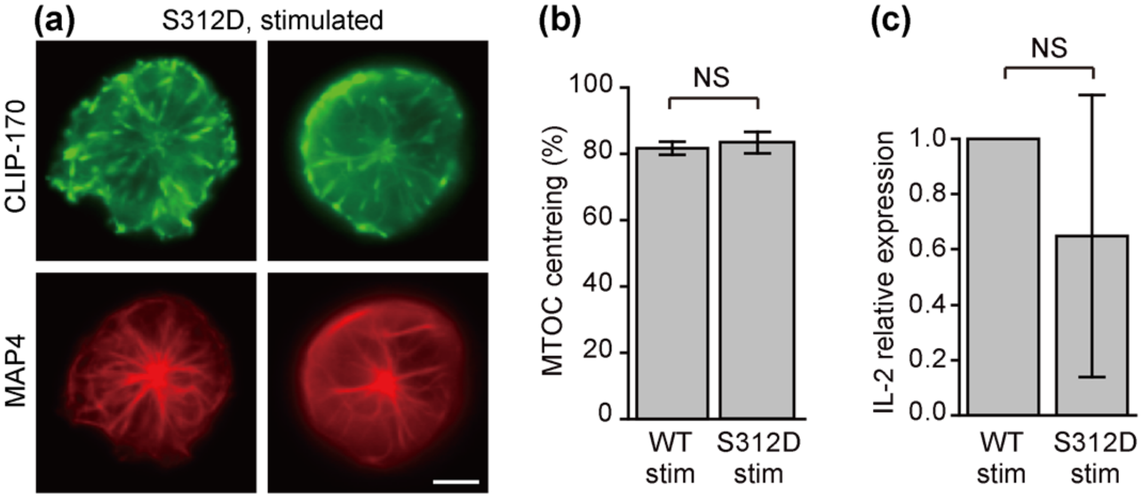

Figure S5. CLIP-170 S312D phosphomimetic mutation does not affect either MTOC repositioning or IL-2 expression. (**a**) Jurkat T cells expressing both mEGFP tagged CLIP-170 S312D and TagRFP-T-MAP4 were stimulated by incubation on the anti-CD3ε/anti-CD28 surface, then the images were captured. Protein distribution was similar to wild type. Bar, 5 μm. (**b**) MTOC centreing of MAP4 was measured as described in Figure S2. (**c**) The total RNA was extracted from mEGFP tagged CLIP-170 S312D expressing Jurkat T cells, and the relative IL-2 expression was quantified by qPCR.

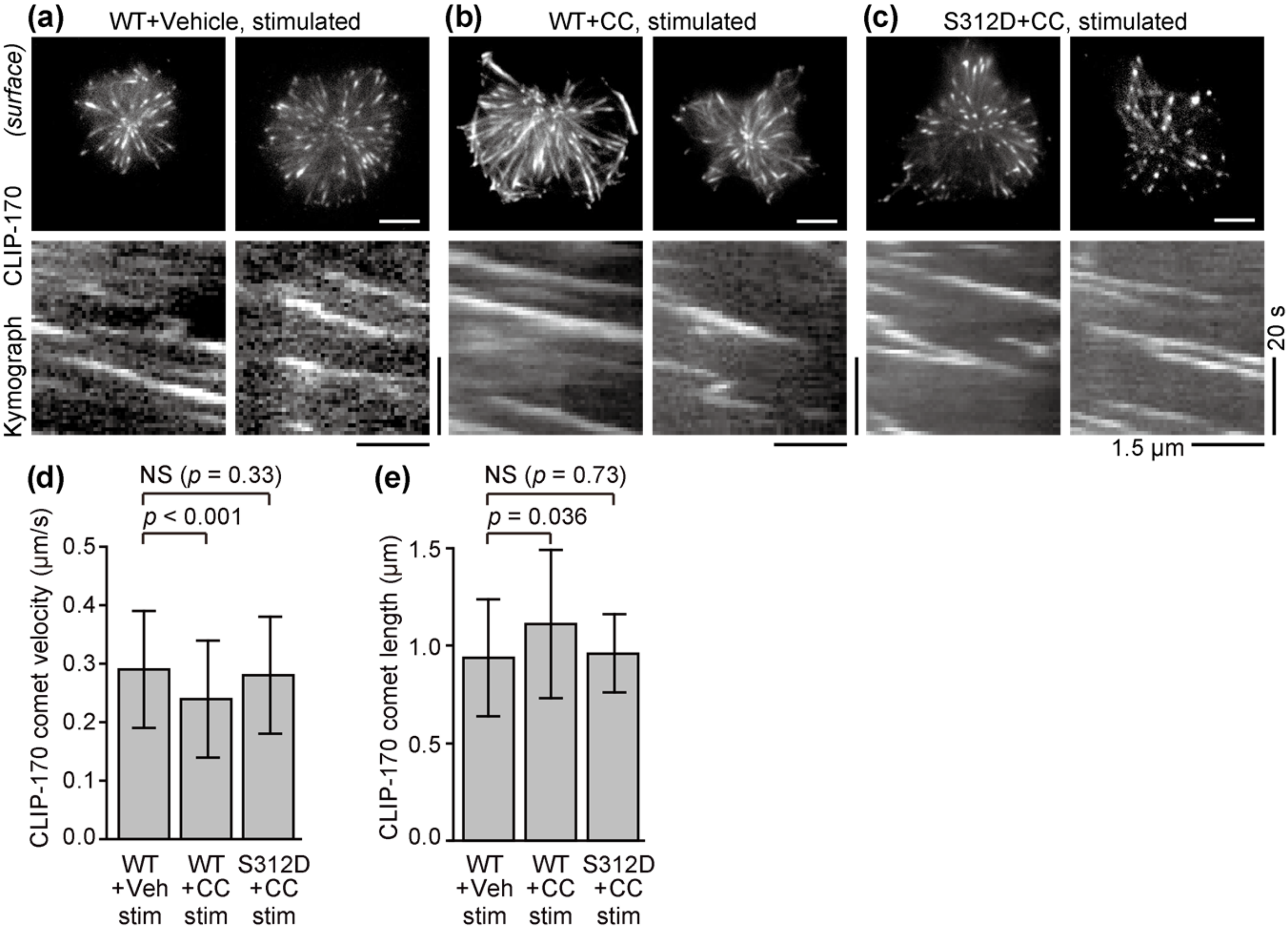

Figure S6. CLIP-170 phosphorylation at Ser-312 by AMPK up-regulates the microtubule plus-end dynamics. (**a-c**) TIRF live-cell images (top) and kymograph (bottom) of CLIP-170 comets and microtubule plus-end dynamics visualized using mEGFP-tagged CLIP-170s: wild-type in the presence of vehicle alone (0.2% DMSO, **a**) or 20 μM compound C (AMPK inhibitor, **b**), and phosphomimetic S312D mutant in the presence of 20 μM compound C (**c**) (See Movie S2). (**d, e**) The velocity (**d**) and the length (**e**) of CLIP-170 comets quantified using fluorescence images. Jurkat cells were stimulated with the anti-CD3ε/anti-CD28 antibodies coated on glass bottom dishes. Scale bar in TIRF images, 5 μm; horizontal and vertical scale bars in kymographs, 1.5 μm and 20 s, respectively. Data are means ± SD.

Supplementary Tables

Table S1. The MTOC distance from the cell surface of contact (source data for Figs. 1c,d & 2i,j)

	MTOC distance z from the contact surface[a] (μm)	MTOC centreing (%)	N_{cell}
WT (stimulated)	0.94 ± 0.31	79.2 ± 7.2	37
WT (unstimulated)	2.14 ± 1.26	20.4 ± 14.9	25
S312A (stimulated)	1.15 ± 0.59	28.2 ± 8.1	25

Data are mean ± standard deviation (SD). N_{cell} indicates the number of cells.

[a] The MTOC distance z from cell surface was corrected using Equation 1.

Table S2. The fraction of MTOC centreing and IL-2 relative expression (source data for Fig. 1g,h & 1k,l)

	MTOC centreing (%)	N_{cell}	IL-2 relative expression[a]
shControl (stimulated)	60.7 ± 3.0	37	1 [b]
shCLIP-170 (stimulated)	46.5 ± 7.5	43	0.72 ± 0.1
Vehicle (stimulated)	80.9 ± 5.2	47	1 [b]
CC (stimulated)	42.3 ± 11.5	52	0.49 ± 0.1

Data are mean ± SD. N_{cell} indicates the number of cells.

[a] The relative expression of IL-2 was quantified by qPCR in triplicate.

[b] Normalised to the data from this reference gene.

Table S3. The fraction of MTOC centreing and IL-2 relative expression (source data for Fig. 2c,f,g)

	MTOC centreing (%)	N_{cell}	IL-2 relative expression[a]
WT	81.6 ± 2.0	25	1 [b]
S312A	33.3 ± 9.2	42	0.61 ± 0.1
WT/CC	39.1 ± 1.9	64	
S312D/CC	77.8 ± 1.9	27	

Data are mean ± SD. N_{cell} indicates the number of cells.

[a] The relative expression of IL-2 was quantified by qPCR in triplicate.

[b] Normalised to the data from this reference gene.

Table S4. CLIP-170 comet velocities and lengths (source data for Figs. 3d,e & S6d,e)

	Comet velocity (µm/s)	Comet length (µm)	N_{comet}
WT, stimulated	0.32 ± 0.08	0.99 ± 0.23	30
S312A, stimulated	0.13 ± 0.04	1.10 ± 0.16	30
WT, unstimulated	0.28 ± 0.07	1.03 ± 0.27	30
WT + vehicle, stimulated	0.29 ± 0.06	0.94 ± 0.25	30
WT + CC, stimulated	0.24 ± 0.05	1.11 ± 0.38	30
S312D + CC, stimulated	0.28 ± 0.07	0.96 ± 0.17	30

Data are mean ± SD. N_{comet} indicates the number of comets.

Table S5. The velocities of the clusters of CLIP-170, DLC and dynactin (source data for Fig. 4i-k)

		Velocity (µm/s)			
		[−]	[+]	$N_{cluster}$	Average C and P
WT CLIP-170, not colocalised	C		0.27 ± 0.08 [e]	25	0.28 ± 0.08
stimulated [a]	P		0.30 ± 0.09 [e]	33	
WT CLIP-170 & DLC, colocalised	C		0.15 ± 0.06 [f]	9	0.15 ± 0.07
stimulated [b]	P		0.15 ± 0.08 [f]	7	
WT CLIP-170, not colocalised	C		0.28 ± 0.06 [e]	13	0.27 ± 0.05
unstimulated [b]	P		0.27 ± 0.03 [e]	9	
WT CLIP-170 & DLC, colocalised	C		ND	–	
unstimulated [b]	P		0.16 ± 0.03 [f]	3	
S312A CLIP-170, not colocalised	C		0.20 ± 0.05	15	0.20 ± 0.05
stimulated [c]	P		0.20 ± 0.06	15	
DLC, not colocalised	C	0.057 ± 0.028		22	0.05 ± 0.03
stimulated [b]	P	0.049 ± 0.029		21	
DLC, not colocalised	C	0.058 ± 0.014		3	0.06 ± 0.03
unstimulated [b]	P	0.059 ± 0.032		11	
DLC, not colocalised	C	0.061 ± 0.032		9	0.05 ± 0.03
S312A, stimulated [c]	P	0.051 ± 0.025		29	

dynactin, not colocalised	C		0.27 ± 0.03 [e]	2	0.26 ± 0.03 [e]
stimulated [d]	P		0.26 ± 0.04 [e]	3	
CLIP-170 & dynactin, colocalised	C		0.29 ± 0.08 [e]	13	0.29 ± 0.07
stimulated [d]	P		0.30 ± 0.05 [e]	11	
Average of all [–]		0.054 ± 0.028		95	
Average of [+] [e]			0.28 ± 0.07	109	
Average of [+] [f]			0.15 ± 0.06	19	

Data are mean ± SD. N_{cluster} indicates the number of clusters.

[–] and [+] denote the minus-end-directed movement and plus-end tracking, respectively. WT, S312A, and DLC denote wild-type, phosphodeficient S312A mutant, and dynein light chain, respectively. C and P denote the centre and periphery region, respectively.

[a] Data from TIRF images using Jurkat cells co-expressing DLC-mEGFP and wild-type CLIP-170-TagRFP-T, those co-expressing dynactin-mEGFP and wild-type CLIP-170-TagRFP-T, and those co-expressing CD3ζ-mEGFP and wild-type CLIP-170-TagRFP-T stimulated on antibody-coated dishes.

[b] Data from TIRF images using Jurkat cells co-expressing DLC-mEGFP and wild-type CLIP-170-TagRFP-T stimulated and unstimulated on antibody-coated dishes.

[c] Data from TIRF images using Jurkat cells co-expressing DLC-mEGFP and S312A mutant CLIP-170-TagRFP-T stimulated on antibody-coated dishes.

[d] Data from TIRF images using Jurkat cells co-expressing dynactin-mEGFP and wild-type CLIP-170-TagRFP-T stimulated on antibody-coated dishes.

[e] Average of [+] [e] is the average of the [+] velocities of the followings (indicated by superscript e): WT CLIP-170 not colocalised, stimulated; WT CLIP-170 not colocalised, unstimulated; dynactin not colocalised, stimulated; CLIP-170 & dynactin colocalised, stimulated.

[f] Average of [+] [f] is the average of the [+] velocities of the followings (indicated by superscript f): WT CLIP-170 & DLC colocalised, stimulated; WT CLIP-170 & DLC colocalised, unstimulated.

Table S6. Movement durations of dynein clusters (source data for Fig. 4g-k)

	Movement duration of dynein clusters (s)			
	Minus-end movement [−] [a]	$N_{cluster}$	Plus-end tracking [+] [b]	$N_{cluster}$
Stimulated [c]	16.1 ± 8.4 [e]	43	8.9 ± 3.0 [f]	16
Unstimulated [c]	14.3 ± 7.5 [e]	13	8.3 ± 2.5 [f]	3
Stimulated (S312A) [d]	18.5 ±14.6 [e]	38	ND	–
Average	16.8 ± 11.2 [g]	94	8.8 ± 2.9	19

Data are mean ± SD. $N_{cluster}$ indicates the number of clusters.

[a] All of the minus-end-directed clusters of dynein were not colocalised with CLIP-170 clusters.

[b] All of the plus-end tracking clusters of dynein were colocalised with CLIP-170 clusters.

[c] Data from TIRF images using Jurkat cells co-expressing DLC-mEGFP and wild-type CLIP-170-TagRFP-T stimulated and unstimulated on antibody-coated dishes.

[d] Data from TIRF images using Jurkat cells co-expressing DLC-mEGFP and S312A mutant CLIP-170-TagRFP-T stimulated on antibody-coated dishes.

[e] No significant difference among these three assessed using *t*-tests.

[f] No significant difference between these two assessed using *t*-tests.

[g] This value is an underestimation due to photobleaching.

Table S7. Primers used for plasmid preparations

Primers for CLIP-170	
1	Forward 5’-AGCTGTCGACGCCACCATGAGTATGCTAAAGCCAAGTGGGC-3’
2	Reverse 5’-CAACTGCAATGACGACGAAACCTTCGGGGATCCAGCT-3’
Primers for site-directed mutagenesis of S312A mutant of CLIP-170	
3	Forward 5’-CGCAGCCCTGCTGCCTCTTCCCTCAG-3’
4	Reverse 5’-CTGAGGGAAGAGGCAGCAGGGCTGCG-3
Primers for site-directed mutagenesis of S312D mutant of CLIP-170	
5	Forward 5’-CGCAGCCCTGATGCCTCTTCCCTCAG-3’
6	Reverse 5’-CTGAGGGAAGAGGCATCAGGGCTGCG-3’
Primers for dynein light chain	
7	Forward 5’-AGCTGCTAGCGCCACCATGTGCGACCGAAAGGCCGTGATCA-3’
8	Reverse 5’-AGCTCCGCGGACCAGATTTGAACAGAAGAATGGCC-3’
Primers for dynactin	
9	Forward 5’-AGCTGCTAGCGCCACCATGGCACAGAGCAAGAGGCACGTGT-3’
10	Reverse 5’-AGCTCCGCGGGGAGATGAGGCGACTGTGAAGCTGG-3’

Table S8. Primers for qPCR (quantitative real-time PCR)

Primers for RT-PCR of IL-2 (relative expression of IL-2)	
3	Forward 5’-GCATTGCACTAAGTCTTGCAC-3’
4	Reverse 5’- TCCTGGTGAGTTTGGGATTC-3’
Primers for RT-PCR of β-Actin (control, relative expression of IL-2)	
5	Forward 5’-AGAGCTACGAGCTGCCTGAC-3’
6	Reverse 5’-AGCACTGTGTTGGCGTACAG-3’
Primers for RT-PCR of CLIP-170 (knockdown efficiency)	
1	Forward 5’-CAGTGGGCTGGAATTGTTTT-3’
2	Reverse 5’-GTCTGCAGGCCATTAGCTTC-3’

Table S9. Short hairpin RNAs (shRNAs) used for CLIP-170 knockdown

CLIP-170 shRNA #1	
1	Sense<br>5'-TGCTGCTTAGTGCCACCAACCAATACGTTTTGGCCACTGACTGACGTATTGGTGTGGCACTAAG-3'
2	Antisense<br>5'-CCTGCTTAGTGCCACACCAATACGTCAGTCAGTGGCCAAAACGTATTGGTTGGTGGCACTAAGC-3'
CLIP-170 shRNA #2	
3	Sense<br>5'-TGCTGATCAGCTGCTCTTCTCTCTCAGTTTTGGCCACTGACTGACTGAGAGAGGAGCAGCTGAT-3'
4	Antisense<br>5'-CCTGATCAGCTGCTCCTCTCTCAGTCAGTCAGTGGCCAAAACTGAGAGAGAAGAGCAGCTGATC-3'
Control shRNA	
5	Sense<br>5'-TGCTGAAATGTACTGCGCGTGGAGACGTTTTGGCCACTGACTGACGTCTCCACGCAGTACATTT-3'
6	Antisense<br>5'-CCTGAAATGTACTGCGTGGAGACGTCAGTCAGTGGCCAAAACGTCTCCACGCGCAGTACATTTC-3'
